# Developing and evaluating a SAFER model to screen for diabetes complications among people experiencing homelessness: a pilot study protocol

**DOI:** 10.1186/s40814-022-01165-2

**Published:** 2022-09-16

**Authors:** Sara Scott, Eshleen K. Grewal, Hamna Tariq, David J. T. Campbell

**Affiliations:** 1 Department of Medicine, Cumming School of Medicine, University of Calgary, 3280 Hospital Drive NW, TRW 3E3, Calgary, Alberta T2N 4Z6 Canada; 2 Department of Medicine, Faculty of Medicine & Dentistry, University of Alberta, Edmonton, Alberta Canada; 3 Department of Community Health Sciences, Cumming School of Medicine, University of Calgary, Calgary, Alberta Canada; 4 Department of Cardiac Sciences, Cumming School of Medicine, University of Calgary, Calgary, Alberta Canada

**Keywords:** Point-of-care screening, Diabetes complications, Homelessness, Access to care, Models of care, Patient-centered, Healthcare equity, Health innovation, Pilot study

## Abstract

**Background:**

Diabetes management combined with housing instability intersects, forcing individuals to triage competing needs and critical stressors, such as safety and shelter, with fundamental diabetes self-management tasks like attending healthcare appointments to screen for the complications of diabetes, leaving individuals overwhelmed and overburdened. We aim to address this disjuncture found within our current healthcare delivery system by providing point-of-care screening opportunities in a more patient-centered approach.

**Method:**

We describe a pilot study of a novel clinical intervention which provides timely, comprehensive, and accessible screening for diabetes complications to people experiencing homelessness. We will assess the reach, effectiveness, adoption, implementation, and maintenance, as per the RE-AIM framework, of a SAFER model of care (i.e., screening for A1C, feet, eyes, and renal function). A trained nurse will provide this screening within a homeless shelter. During these encounters, eligible participants will be screened for microvascular complications (neuropathy, nephropathy, retinopathy) and have their A1C measured, all at the point of care, using bedside tools and novel technology. Effectiveness, our primary objective, will be evaluated using a pre-post design, by comparing the rate of completion of full microvascular screening during the study period with individuals’ own historical screening in the 2-year period prior to enrollment. The other domains of the RE-AIM framework will be assessed using process data, chart reviews, patient surveys, and qualitative semi-structured interviews with service providers and participants. This study will be conducted in a large inner-city homeless shelter within a major urban Canadian city (Calgary, Canada).

**Discussion:**

Currently, screening for diabetes complications is often inaccessible for individuals experiencing homelessness, which places heavy burdens on individuals and, ultimately, on already strained emergency and acute care services when complications go undetected at earlier stages. The SAFER intervention will modify the current standard of care for this population in a way that is less fragmented, more person-focused, and timely, with the goal of ultimately improving the rate of screening in an acceptable fashion to identify those requiring specialist referral at earlier stages.

**Supplementary Information:**

The online version contains supplementary material available at 10.1186/s40814-022-01165-2.

## Background

Precarious housing is a major social determinant of health and a contributor to unsustainable healthcare costs. Although difficult to measure, in Canada, it is estimated that 235,000 people experience homelessness annually [1], and up to 22% of these individuals also live with diabetes (approximately 47,000 people in Canada) [2]. These individuals often do not receive the preventative care required to optimally manage their diabetes [3] and are prone to poorer diabetes outcomes compared to the general population managing diabetes [4–6]. They have been described as one of the hardest-to-reach groups for preventative primary care and are much more likely to use costly emergency care services compared to the general population [7, 8]. People with lived experience of homelessness (PWLEH) face significant barriers to accessing healthcare services [7, 9]. These barriers include but are not limited to attending to competing needs, demoralizing anti-homelessness sentiment/biases woven into many institutional settings, financial barriers and lack of healthcare cards or other identification, and scheduling systems which suit providers but are inconvenient for patients [10]. Many patients lack access to phones and means of transportation required to attend medical appointments [7, 11, 12]. The COVID-19 pandemic has exacerbated this issue, causing further barriers to accessing preventative healthcare as programs and providers have either refocused their efforts on responding to acute needs or many medical services have pivoted to remote visits, which has at times caused further accessibility barriers for this group, which is referred to as “the digital divide” [13–15].

Although identifying and addressing the root causes of homelessness and diabetes are top priorities in bolstering the health of communities, timely access to screening that identifies early signs of preventable diabetes-related complications is also fundamental to improving outcomes in this population and should not be ignored [16–19]. Comprehensive screening includes regular screening of the retina (retinal fundoscopy) to look for early signs of diabetic retinopathy [16], the feet (standardized diabetic foot assessments) to look for early signs of diabetic neuropathy and peripheral vascular disease [17], and the kidneys (serum creatinine and albuminuria [ACR] test) to look for early signs of diabetic nephropathy [18]. Furthermore, monitoring glycosylated hemoglobin levels (A1C) every 3-6 months is recommended, as this guides self-management and treatment decisions. Maintaining blood glucose levels in the optimal range (A1C < 7%) has a global effect on reducing diabetes-related complications including those mentioned above [19]. Once complications are detected, referral to subspecialists for intervention can reduce permanent morbidity (blindness, kidney failure, amputations). Despite the importance of this regular screening, one recent prospective study found that among PWLEH with diabetes, only 12–30% met the annual screening targets for microvascular complications [19]. Barriers identified to screening completion for PWLEH include scheduling and logistical issues (such as difficulty making appointments) and lack of control over their time [20, 21].

A core pillar of the *Canada Health Act* is accessibility [22]. This is in place to ensure residents of Canada have reasonable access to medically necessary services without financial barriers [22]. Despite this fact, there are numerous inequities in Canadian health care delivery, resulting in some segments of the population still facing barriers to accessing medical care [23]. There is an opportunity for healthcare systems to re-center their approach towards innovative, effective, and equitable strategies that better align with the Equity to Access in Medical Care Framework [24] which was recently validated in PWLEH [25]. This framework articulates that to have equitable utilization of health services and positive consumer experiences, supportive health policies need to be in place, health delivery systems need to promote access and continuity of care, and the specific needs and context of populations must be considered in the implementation of such services, with the psychological safety of users as a top priority.

Facilitating screening and subsequent referral to specialists, tailored to the complex needs of PWLEH, has the potential to reduce the incidence of advanced diabetes-related complications and improve health outcomes in a cost-effective way for this population. Without the opportunity and support to optimally manage one’s diabetes, experiences of homelessness are further complicated, threatening employment capacity, increasing chronic stress, and adding to the disease burden one carries. While the need for non-traditional, forward-thinking, and equitable healthcare delivery models for PWLEH is frequently recommended in the literature [6, 7, 9, 26], less is known about the process to enact this, especially as related to screening for diabetes complications. Within our pilot study, we will explore a novel approach that strives to enable PWLEH to complete comprehensive screening for diabetes complications within familiar settings and, subsequently, connect PWLEH to subspecialty follow-up support as required, while prioritizing patient centricity throughout the process.

## Methods

### Study design

We will use a concurrent convergent mixed-methods approach to assess the Reach, Effectiveness, Adoption, Implementation, and Maintenance (RE-AIM) [27] of a novel point-of-care screening model we have named SAFER (screening A1C, feet, eyes, and renal), for diabetes microvascular complications in those who are experiencing homelessness.

### Setting

This pilot study will take place in Calgary, Canada, between January 2022 and December 2022. Calgary is a metropolitan city of 1.3 million residents [28], with a sizeable population of people experiencing homelessness: nearly 3000 on a given night, as per the 2018 point-in-time count [29]. Participants will be recruited through the Calgary Drop-In Centre, which is Canada’s largest homeless shelter, offering both emergency shelter and many programs focused on supporting clients to achieve permanent housing [30]. In Canada, medically necessary healthcare services provided by physicians and some allied health professionals are covered by provincial health insurance plans and provided at no cost to individual patients. Providers can bill the government payer for these services rather than billing patients directly [31]. Generally, the screening tests required for diabetes are covered under these plans. However, patients still face barriers as these services are not tailored to the needs of PWLEH and often require individuals to visit multiple locations to receive this care, including primary care provider office, separate laboratory location, ophthalmology/optometry clinics, and foot care nurse or podiatrist [32]. When a complication is detected, referrals to specialists are often made, and while patient payment for these is also not required, this necessitates visiting other locations to which PWLEH face another set of significant barriers, including lack of control over one’s time, and logistical barriers such as limited abilities to arrange appointments and transportation as discussed earlier [20].

### Intervention

**Fig. 1:**
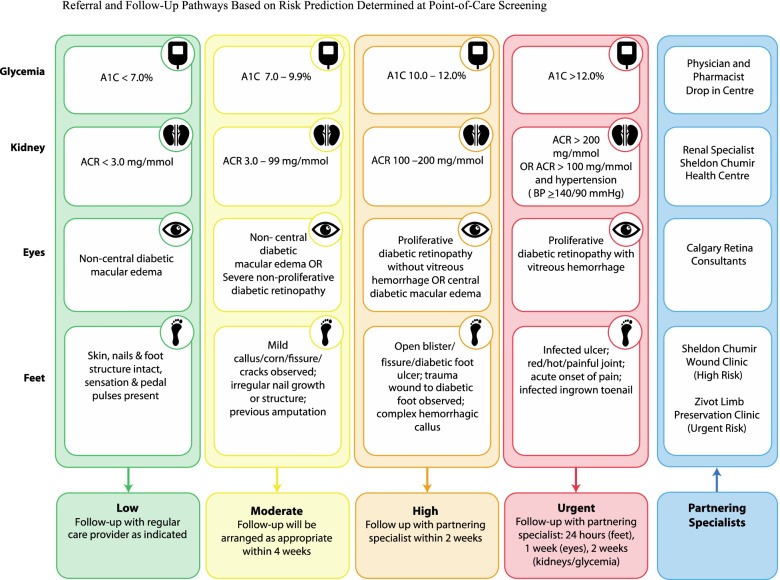
Pathways

A trained clinical research nurse (CRN) will perform all screening maneuvers and make necessary referrals to partnering specialists based on screening results, using predetermined care pathways created in collaboration with local specialists (see Fig. 1). The intervention consists of the following screening steps:

1. Participant to obtain a urine sample for the CRN.
  Urine samples will be provided to determine the albumin-to-creatinine ratio (ACR) to detect abnormal protein levels in the urine as an early marker of renal impairment. We will analyze the urine sample at the point of care using the Siemens DCA Vantage Analyzer [33, 34]. If the ACR reading is above 20 mg/mmol (which is the limit of detection for this device), the remaining urine specimen will be sent to a laboratory to confirm the value, and additional blood work will be taken by the partnering facility staff in order to prepare for a renal specialist consult. This will eliminate the need for participants to make a laboratory appointment prior to their follow-up visit.
2. The CRN will perform a visual acuity check, intraocular pressure reading, and dilate the pupils in preparation for the retinal image screen.

- Visual acuity checks will be completed with a 10-ft Snellen visual acuity wall chart.
- Intraocular pressure will be measured using a minimal contact portable tonometer [35].
- The CRN will explain the need to dilate both pupils in order to complete the retinal screen. Dilation drops (tropicamide 1% + phyenylephrine 2.5%) will be applied and allowed to reach full effect over the next 15 min.

3. The CRN will collect a capillary blood sample (5 μL) via finger prick, which will be tested using the Siemens DCA Vantage Analyzer for glycosylated hemoglobin (A1C) [33].
4. The CRN will check the patient’s vital signs, specifically blood pressure and heart rate.
5. Once the pupils have had time to dilate, the CRN will capture retinal fundus images using a specialized non-mydriatic retinal camera [36] aiming to capture fields 0, 1, 2, 3, 6, and 7 of the retina. The photos will then be uploaded to a secure database where our partnering ophthalmologist will review and provide a comprehensive report on the presence and status of diabetic retinal complications, including intraretinal hemorrhage, macular edema, microaneurysms, and exudates.
6. The CRN will perform a comprehensive neurovascular diabetic foot examination following the Alberta Health Services Foot Screen Tool [37]. The CRN will then provide basic foot care: wash feet; trim toenails; remove uncomplicated calluses, corns, and ingrown toenails; clean and dress any basic wounds; and moisturize the feet.
7. Depending on the results obtained, the CRN will send referrals to partnering specialists based on risk prediction (see Fig. 1).
8. At the follow-up visit, the CRN will share the screening report with the participant and partnering facility medical staff for their medical records and will assist in facilitating follow-up appointments with any necessary specialists (see Additional file 2: Appendix A).

### Objectives

Our primary objective, focused on effectiveness, is to evaluate the impact of point-of-care screening for diabetes complications on the rate of completion of full microvascular screening (eyes, kidneys, feet) and glycemic monitoring (A1C), compared to usual care, among PWLEH and diabetics. Specific objectives are listed in Table 1.

**Table 1:** Reach, Effectiveness, Adoption, Implementation, and Maintenance (RE-AIM) areas of inquiry of the SAFER protocol pilot trial

Reach	Effectiveness	Adoption	Implementation	Maintenance
How many participants were willing to participate in the study? How many people declined participation in the study? *Data sources: consent to contact forms and qualitative interviews with service providers*	**Primary objective: What is the incremental increase in the completion of comprehensive screening and glycemic monitoring?** *Data sources: chart review, participant clinical record*	What challenges did facilities face in the process of implementing the intervention? *Data sources: qualitative interviews with service providers*	Was the intervention delivered consistently? *Data sources: field notes*	What were the barriers to continuing this program after completion of the pilot trial, as described by service providers? *Data sources: qualitative interviews with service providers*
Of those who agreed to participate, how many attended both the baseline and the follow-up sessions? *Data sources: participant clinical record*	How many participants with complications, detected as a result of the screening intervention, were referred to a specialist for follow-up care? *Data sources: participant clinical record and field notes*	According to both participants and providers, was the intervention well received? *Data sources: qualitative interviews with service providers and participants*	Was it feasible to deliver the intervention in the allotted time frame? *Data sources: field notes*	According to the participants, would they continue attending the screening intervention on a yearly basis if it became a permanent program? *Data sources: qualitative interviews with participants*
What were the clinical and sociodemographic characteristics of the participants? *Data sources: baseline questionnaire and participant clinical record*	How many participants attended specialist referral appointments? *Data sources: participant clinical record and field notes*		Was the intervention delivered as intended? *Data sources: field notes*	Did partnering facilities continue to deliver the intervention after the pilot trial was complete? Why or why not? *Data sources: qualitative interviews with service providers*
	What was the incremental increase in specialist visits for participants requiring follow-up care? *Data sources: chart review, participant clinical record*		How many participants attended both visits with the CRN? *Data sources: Participant clinical record*	What proportion of service providers indicated a willingness to deliver the intervention after the conclusion of the pilot trial? What were their motivations for wanting to do that? *Data sources: qualitative interviews with service providers*
	What were the participants’ experiences with the program? *Data sources: qualitative interviews with participants*			

#### Reach

Reach [38] is the number, proportion, and representativeness of the participants who took part in the project. We will assess the reach by answering the following questions and using descriptive statistics:

1. How many eligible individuals participated in the study? How many eligible people declined participation in the study?
2. Of those who agreed to participate, how many attended both the baseline and the follow-up sessions?
3. What were the clinical and sociodemographic characteristics of the participants?

#### Effectiveness

The effectiveness [39] of the screening intervention will be determined by assessing the impact of the intervention on various outcomes. The following questions will be used to assess the impact:

1. What is the incremental increase in the completion of comprehensive screening and glycemic monitoring for each individual, compared to the 2 years prior? [*primary outcome*]
2. How many participants with complications, detected as a result of the screening intervention, were referred to a specialist for follow-up care?
3. How many participants attended their specialist referral appointments?
4. What was the incremental increase in specialist visits for participants following the intervention, compared to 2 years prior?
5. What were the participants’ experiences with the program?

#### Adoption

Adoption [40] refers to the number, proportion, and representativeness of providers at partnering facilities who would be willing to implement the screening intervention after the pilot study, and it includes their reasons for wanting to do so. It will be evaluated by pursuing answers to the following questions and using qualitative analysis approaches:

1. What challenges did the facility face in the process of implementing the intervention?
2. According to both participants and providers, was the intervention well received?

#### Implementation

The implementation [41] of the intervention includes determining whether the intervention was consistently delivered as intended, taking into account the intended time and cost and the adjustments that were made during delivery. The following specific questions will be answered to assess the implementation:

1. Was the intervention delivered consistently?
2. Was it feasible to deliver the intervention in the allotted time frame?
3. Was the intervention delivered as intended (i.e., fidelity)?
4. How many participants attended both visits with the CRN?

#### Maintenance

Maintenance [42] concerns whether the intervention tested in this pilot has the potential to become part of routine practices and policies at partnering facilities. It also refers to the long-term effects of the intervention outcomes, once the study is complete. For maintenance, we will consider the following specific objectives and use a combination of descriptive statistics and qualitative analysis approaches:

1. What are the barriers to continuing this program after completion of the pilot trial, as described by service providers and management?
2. Would participants continue attending the screening intervention on a yearly basis if it became a permanent program?
3. Did the partnering facilities continue to deliver the intervention after the pilot was complete? Why or why not?
4. What proportion of providers indicated a willingness to deliver the intervention after the conclusion of the pilot trial? What were their motivations for wanting to do that?

### Theoretical framework

Our intervention is informed by the Minimally Disruptive Medicine model which advocates for a patient-centered approach to clinical care that focuses on acknowledging the difficulties and complexities of managing a chronic disease in the context of other co-morbidities and social stressors [43]. It is designed to place the least possible burden on a patient by identifying and addressing the possible barriers to optimal care and incorporates an individualized approach to shared decision-making. Our intervention is informed by this model and principles in that we are attempting to minimize the burden placed upon people in seeking required screening for diabetes complications.

### Sampling and recruitment

Given that this is a pilot study, a firm sample size is not required. That being said, we are aiming to recruit at least 50 participants. While this sample size is imprecise, we are hoping to recruit as many participants as possible. At least 50 participants will allow us to have a sense of the suitability of this program and for a full-scale trial.

All participants will be recruited through the Drop-In Centre (the DI). The DI will advertise a weekly point-of-care diabetes complications screening clinic for individuals accessing the facility’s services through posters and word of mouth. Due to infection control measures initiated by the pandemic, this pilot was initially open to residents of the DI only. However, as we have returned to pre-pandemic protocols, the inclusion criteria have expanded to adults who access DI services but may not be current residents of the DI.

Ideally, three to four participants are seen within each clinic. The CRN will have the ability to see *up to five* participants within each clinic to accommodate a mix of initial and follow-up visits and will be flexible on the day of in response to participant needs and availability. Each participant is asked to attend an initial screening visit (just over 60 min in length) and a second follow-up visit (approximately 45 min in length). Ideally, both visits are prebooked in collaboration with the DI medical staff; however, the research team understands that plans can change suddenly. The DI medical staff will remind participants in person of the upcoming appointments and will call if they have a phone to provide reminders leading up to the clinic. If a participant is late for their appointment, the DI will overhead page participants or walk around the facility to look for them to provide a friendly reminder. To maximize efficiency, the research team will keep a running to-do list ready in case of last-minute cancelations and will pivot to walk-in appointments, follow-up appointments over the phone, or other logistical tasks involved with the project, ensuring no time is wasted when cancelations do occur.

To participate in the pilot, participants must meet the following inclusion and exclusion criteria:

The following are the inclusion criteria:

1. Adults (age 18–85 years)
2. Currently experiencing homelessness, defined according to three out of the four classifications outlined by the Canadian Observatory on Homelessness [44] including: (1) individuals who are unsheltered (or rough sleeping), (2) those who are utilizing emergency shelters, and (3) those who are provisionally accommodated [40].
3. Self-reported diagnosis of diabetes mellitus (any type)
4. Can communicate in English or have someone to translate for them
5. Residing within the DI or directly using services at the DI
6. Willing to spend up to 2 h with the research team on screening and data collection activities

The following are the exclusion criteria:

1. Intoxicated during the screening visit
2. Uncontrolled mental illness that precludes screening assessments (acute psychosis or active mania)
3. Individual already has comprehensive diabetes specialist care, including consistent access to *all* of the following: primary care provider/diabetes specialist, ophthalmologist/optometrist, nephrologist/kidney care team, and foot care nurse/podiatrist

### Data collection

There are two phases of the data collection process for this study, which are outlined below.

#### Initial visit

1. After the completion of informed consent, the research associate (RA) will hand baseline surveys to the participant. The RA will read the questions to the participant if the participant agrees.
  The following surveys will be administered to collect research data during the initial visit (Additional file 3: Appendix B):

- Baseline Demographic Questionnaire
- Diabetes Complication Screening Questionnaire: Baseline
- The Diabetes Self-Management Questionnaire (DSMQ) [45]
- The Problem Areas in Diabetes Scale (PAID-5) Questionnaire [46]

2. The CRN will then complete the screening and record the screening values in the participant’s research record (as described above).
3. The RA will then book the follow-up appointment with the participant and provide the first $10 CAD honorarium.
4. The CRN will complete data entry and make referrals as required based on the referral pathways (Fig. 1).

#### Follow-up visit

The follow-up visit will occur 1 to 2 weeks post-intervention.

1. The CRN will provide the follow-up screening report (Additional file 2: Appendix A) to the participant at the second visit, with specialist appointments and arrangements made as required. She/he will answer any questions the participant has at that time regarding ongoing clinical management.
2. The RA will then conduct the post-intervention survey and semi-structured interview (Additional file 4: Appendix C and Additional file 5: Appendix D).
3. The RA will provide basic education on diabetes and its complications. The basic education provided is shared verbally by the RA based on Diabetes Canada recommendations for diabetes-related complication screening [47] and in one short video [48] right before the closing thoughts portion of the interview (Additional file 5: Appendix D).
4. The RA will also highlight the recommended screening intervals at the bottom of the participant report (Additional file 2: Appendix A).
5. The CRN will provide the partnering facility with the participant’s report and care plan for them to add to their respective charting system.
6. The RA will provide the participant with the final study honorarium ($20 CAD).

### Data management and analysis

Qualtrics survey software (Seattle, WA) will be used to manage the survey data collected. The participants will complete the surveys in Qualtrics, and their responses will be stored in the online software. Our primary objective, to evaluate the impact of point-of-care screening for diabetes complications on the rate of completion of full microvascular screening and glycemic monitoring, compared to usual care, will be dichotomous and, thus, will be analyzed using Fisher’s exact test. We will also use the same approach to determine whether the incremental increase in specialist visits was statistically significant. All other quantitative data will be reported descriptively. All statistical analyses will be conducted using the Stata statistical software [49].

The interviews will be audio-recorded and transcribed, and transcriptions will be organized using the NVIVO™ qualitative data analysis software (QSR International, Melbourne, Australia) and analyzed using a thematic analysis approach, as described by Braun and Clarke [50]. The analysis process will follow all six phases in an inductive nature. For phase 1, we will familiarize ourselves with the data via repeated reading of the transcripts. In phase 2, we will develop initial codes. Phase 3 will require further analysis of these initial codes to develop potential themes. During phase 4, we will review and refine the themes. In phase 5, we will define and name the themes. Finally, for phase 6, we will prepare a report of our findings for dissemination. Two team members (CRN and RA) will conduct the analysis independently. Regular meetings will be held between both team members and at times the greater research team to debrief regarding the emerging findings while progressing through the aforementioned phases.

## Ethical considerations

The research staff have received training in non-violent crisis management as well as additional training offered through the Calgary Distress Centre on mental health, grief and loss, and crisis intervention to better support participants from a psychosocial perspective. The CRN has also received additional training in equity-oriented health care [51] and the Educating for Equity (E4E) Care framework [52], primed with awareness and sensitivity to violence and trauma many people (Indigenous people in particular) experience while accessing healthcare within Canadian contexts. We have developed protocols to support the research team in dynamic clinical settings such as a distress protocol to support participants in distress, safety protocols for values outside of normal clinical ranges, and an acute angle closure glaucoma protocol recommended for all teleophthalmology programs and for all patients receiving mydriatic drops [32].

## Discussion

Individuals navigating diabetes and homelessness do not currently have ideal access to the preventative healthcare services they require, including screening for diabetes complications. The current literature indicates that this population is hard to reach and has many competing needs when considering accessing medical support, yet forward thinking solutions to this problem are rarely studied rigorously. Our SAFER point-of-care screening protocol represents a solution to the abovementioned gap in clinical care provision. We posit that when point-of-care screening is properly implemented, benefits include improved access to comprehensive healthcare, which can lead to improved healthcare partnerships and relationships addressing the lack of trust, common in this population. The design of this novel program may also have applicability to the management of other chronic illnesses among this population. Finally, we will use the learnings from this study to expand our understanding of the SAFER intervention through a full-scale trial.

Despite our best efforts, the proposed study protocol has several limitations. Our point-of-care screening bedside technology does not allow us to determine serum creatinine levels which would provide us with a glomerular filtration rate (GFR) value. This value is also a widely used marker for identifying renal impairment and would provide a more comprehensive assessment for kidney specialists [18, 53]. Unfortunately, we are not aware of any technology that supports this capability at the point-of-care at this time. However, given that the vast majority of diabetic kidney disease begins with an increase in albumin excretion, we should be able to detect most diabetic nephropathy with our albuminuria screen. Future research could support the innovation and development of bedside serum creatinine analysis. Our pilot study does not have a true control group and uses individuals’ historic records as the comparator group. This is prone to recall bias as medical records are likely to be incomplete. If the pilot is successful, a full-scale prospective evaluation will be conducted to overcome this limitation using a pretest-posttest control group experimental design [54] involving multiple sites within Alberta. This will provide more robust evidence of the effectiveness and cost-effectiveness of this model of care. This prospective trial will hypothesize that compared to the control group, the group that receives the SAFER intervention will have better diabetes complication screening rates and report better connections to specialists relevant to their individual health care needs, compared to the control group. We also plan to take a deeper look at the cost-effectiveness of this intervention by building a detailed cost-benefit analysis of this model of care.

## Conclusion

The SAFER intervention described here is expected to improve the quality of care provided, prioritizing patient centricity throughout the process by opportunistically completing screening at the point of care and using more accessible referral pathways to assist patients in receiving necessary care. Completion of these screening procedures has been shown to be related to decreased progression of diabetes-related complications. The results from the evaluation of our pilot will help us determine what works (or does not work), for whom, and why. This will provide new knowledge to inform the design of a future full-scale prospective evaluation of this intervention, which will be refined through this pilot.

## Supplementary Information

**Additional file 1.** CONSORT checklist of information to include when reporting a pilot trial.

**Additional file 2: Appendix A**. Participant report.

**Additional file 3: Appendix B**. Baseline Questionnaire.

**Additional file 4: Appendix C**. Follow-Up Questionnaire.

**Additional file 5: Appendix D**. Interview Guides.

**Additional file 6: Appendix E**. Interview Guide – Health Care Providers.

## Abbreviations

A1C: Glycosylated hemoglobin levels
ACR: Albumin-to-creatinine ratio (mg/mmol)
CRN: Clinical research nurse
GFR: Glomerular filtration rate (mL/min/1.73 m^2^)
DI: Drop-In Centre
DSMQ: Diabetes Self-Management Questionnaire
PAID-5: Problem Area in Diabetes Scale – 5 Items
PWLEH: People with lived experience of homelessness
RA: Research associate
RE-AIM: Reach, Effectiveness, Adoption, Implementation, and Maintenance
SAFER: Screening for A1c, feet, eyes, and renal health
