# Supplementary material for: Developing and evaluating a SAFER model to screen for diabetes complications among people experiencing homelessness: a pilot study protocol
Source: Pilot Feasibility Stud. 2022 Sep 16;8:211. doi: 10.1186/s40814-022-01165-2 (PMC9479401; doi:10.1186/s40814-022-01165-2)
Supplement: Supplementary file 1 — Additional file 1. CONSORT checklist of information to include when reporting a pilot trial. [file 40814_2022_1165_MOESM1_ESM.docx]

| **Additional File 1 - CONSORT checklist of information to include when reporting a pilot trial** | | | | | | |
| --- | --- | --- | --- | --- | --- | --- |
| *Section/topic and item No* | | *Standard checklist item* | | *Extension for pilot trials* | *Page No. where item is reported* | |
| **Title and abstract** | |  | |  |  | |
| 1a | | Identification as a randomised trial in the title | | Identification as a pilot or feasibility randomised trial in the title | 1 | |
| 1b  **Introduction** | | Structured summary of trial design, methods, results, and conclusions (for specific guidance see CONSORT for abstracts) | | Structured summary of pilot trial design, methods, results, and conclusions (for  specific guidance see CONSORT abstract extension for pilot trials) | 2/3 | |
| Background and objectives: | |  | |  | 4-7 | |
| 2a | | Scientific background and explanation of rationale | | Scientific background and explanation of rationale for future definitive trial, and reasons for randomised pilot trial | 4-7 | |
| 2b **Methods** | | Specific objectives or hypotheses | | Specific objectives or research questions for pilot trial | 9-12 | |
| Trial design: | |  | |  |  | |
| 3a | | Description of trial design (such as parallel, factorial) including allocation ratio | | Description of pilot trial design (such as parallel, factorial) including allocation ratio | 6 | |
| 3b | | Important changes to methods after trial commencement (such as eligibility criteria), with reasons | | Important changes to methods after pilot trial commencement (such as eligibility criteria), with reasons | 15 | |
| Participants: | |  | |  |  | |
| 4a | | Eligibility criteria for participants | |  | 12/13 | |
| 4b | | Settings and locations where the data were collected | |  | 7 | |
| 4c | |  | | How participants were identified and consented | 13 | |
| Interventions: | |  | |  |  | |
| 5 | | The interventions for each group with sufficient details to allow replication, including how and when they were actually administered | |  | 7 | |
| Outcomes: | |  | |  |  | |
| 6a | | Completely defined prespecified primary and secondary outcome measures, including how and when they were assessed | | Completely defined prespecified assessments or measurements to address each pilot trial objective specified in 2b, including how and when they were assessed | NA | |
| 6b | | Any changes to trial outcomes after the trial commenced, with reasons | | Any changes to pilot trial assessments or  measurements after the pilot trial commenced, with reasons | NA | |
| 6c | |  | | If applicable, prespecified criteria used to judge whether, or how, to proceed with future definitive trial | NA | |
| **CONSORT checklist of information to include when reporting a pilot trial (Continued)** | | | | | | |
| *Section/topic and item No* | *Standard checklist item* | | *Extension for pilot trials* | | | *Page No where item is reported* |
| Sample size: |  | |  | | |  |
| 7a | How sample size was determined | | Rationale for numbers in the pilot trial | | | 12 |
| 7b | When applicable, explanation of any interim analyses and stopping guidelines | |  | | |  |
| Randomisation: |  | |  | | |  |
| Sequence generation: |  | |  | | |  |
| 8a | Method used to generate the random allocation sequence | |  | | | NA |
| 8b | Type of randomisation; details of any restriction (such as blocking and block size) | | Type of randomisation(s); details of any restriction (such as blocking and block size) | | | NA |
| Allocation concealment mechanism: |  | |  | | |  |
| 9 | Mechanism used to implement the random allocation sequence (such as sequentially numbered containers), describing any steps taken to conceal the sequence until interventions were assigned | |  | | | NA |
| Implementation: |  | |  | | |  |
| 10 | Who generated the random allocation sequence, enrolled participants, and assigned participants to interventions | |  | | | NA |
| Blinding: |  | |  | | |  |
| 11a | If done, who was blinded after assignment to interventions (eg, participants, care providers, those assessing outcomes) and how | |  | | | NA |
| 11b | If relevant, description of the similarity of interventions | |  | | | NA |
| Analytical methods: |  | |  | | |  |
| 12a | Statistical methods used to compare groups for primary and secondary outcomes | | Methods used to address each pilot trial  objective whether qualitative or quantitative | | | 15/16/  22/23 |
| 12b | Methods for additional analyses, such as subgroup analyses and adjusted analyses | | Not applicable | | | NA |
| Recruitment: |  | |  | | |  |
| 14a | Dates defining the periods of recruitment and follow-up | |  | | | 27/28 |
| 14b | Why the trial ended or was stopped | | Why the pilot trial ended or was stopped | | | NA |
| Baseline data: |  | |  | | |  |
| 15 | A table showing baseline demographic and clinical characteristics for each group | |  | | |  |
| Numbers analysed: |  | |  | | |  |
| 16 | For each group, number of participants (denominator) included in each analysis and whether the analysis was by original assigned groups | | For each objective, number of  participants (denominator) included in each analysis. If relevant, these numbers should be by randomised group | | | NA |
| Outcomes and estimation: |  | |  | | |  |
| 17a | For each primary and secondary outcome, results for each group, and the estimated effect size and its precision (such as 95% confidence interval) | | For each objective, results including expressions of uncertainty (such as 95% confidence interval) for any estimates. If relevant, these results should be by randomised group | | | NA |
| 17b | For binary outcomes, presentation of  both absolute and relative effect sizes is recommended | | Not applicable | | | NA |
| Ancillary analyses: |  | |  | | |  |
| 18 | Results of any other analyses performed, including subgroup analyses and adjusted analyses, distinguishing prespecified from exploratory | | Results of any other analyses performed that could be used to inform the future definitive trial | | | NA |
| Harms: |  | |  | | |  |
| 19 | All important harms or unintended effects in each group (for specific guidance see CONSORT for harms) | |  | | | NA |
| 19a **Discussion** |  | | If relevant, other important unintended consequences | | | NA |
| Limitations: |  | |  | | |  |
| 20 | Trial limitations, addressing sources of potential bias, imprecision, and, if relevant, multiplicity of analyses | | Pilot trial limitations, addressing sources of potential bias and remaining uncertainty about feasibility | | | 17 |
| Generalisability: |  | |  | | |  |
| 21 | Generalisability (external validity, applicability) of the trial findings | | Generalisability (applicability) of pilot  trial methods and findings to future definitive trial and other studies | | | 17/18 |
| Interpretation: |  | |  | | |  |
| 22 | Interpretation consistent with results, balancing benefits and harms, and considering other relevant evidence | | Interpretation consistent with pilot trial objectives and findings, balancing potential benefits and harms, and considering other relevant evidence | | | 17 |
| 22a |  | | Implications for progression from pilot to future definitive trial, including any  proposed amendments | | | 17/18 |
| **Other information:** |  | |  | | |  |
| Registration: |  | |  | | |  |
| 23 | Registration number and name of trial | | Registration number for pilot trial and name of trial registry | | | NA |
| Protocol: |  | |  | | |  |
| 24 | Where the full trial protocol can be accessed, if available | | Where the pilot trial protocol can be accessed, if available | | | NA |
| Funding: |  | |  | | |  |
| 25 | Sources of funding and other support (such as supply of drugs), role of funders | |  | | | NA |
| 26 |  | | Ethical approval or approval by research review committee, confirmed with reference number | | | 18/19 |
