# Supplementary material for: Developing and evaluating a SAFER model to screen for diabetes complications among people experiencing homelessness: a pilot study protocol
Source: Pilot Feasibility Stud. 2022 Sep 16;8:211. doi: 10.1186/s40814-022-01165-2 (PMC9479401; doi:10.1186/s40814-022-01165-2)
Supplement: Supplementary file 2 — Additional file 2: Appendix A. Participant report. [file 40814_2022_1165_MOESM2_ESM.docx]

**Appendix A – Participant report**


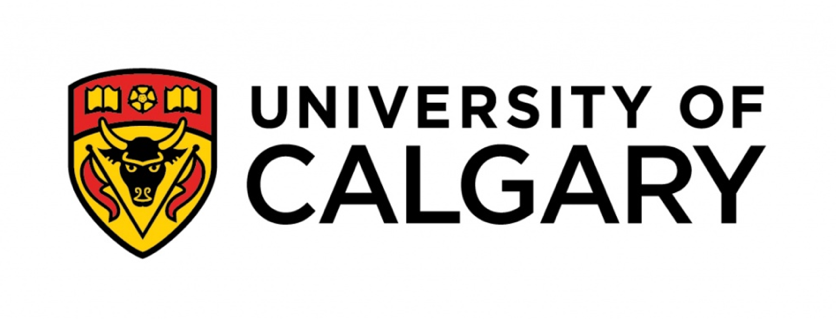


Participant Name:

Point-of-Care Follow Up Date:

Point-of-Care Initial Visit Date:

| Screen: | Result | Suggested Action | Your Follow Up Care Provider |
| --- | --- | --- | --- |
| Eyes |  | *Look for opportunities to get your eyes checked at least once a year by a healthcare provider |  |
| Kidneys | ACR: mg/mmol  ACR results above 2.0 mg/mmol require follow up | *Look for opportunities to get your kidneys checked at least once a year by a healthcare provider |  |
| Blood Sugar | A1C: %  A1C results above 7.0% require follow up | *Look for opportunities to get your blood sugar checked at least every 3 months by a healthcare provider |  |
| Feet |  | *Look for opportunities for foot care maintenance (nail trimming) every 4 weeks  *Inspect your feet daily, keep them dry and let the DI medical staff know if you notice any changes to your feet  *Try to get your feet checked at least once a year by a healthcare provider |  |
| Blood Pressure | mmHg | *Have your healthcare provider continue to check your blood pressure at least once a year |  |

Follow Up Appointments:

Transportation Arrangements:

Your Notes & Questions
