# Supplementary material for: Developing and evaluating a SAFER model to screen for diabetes complications among people experiencing homelessness: a pilot study protocol
Source: Pilot Feasibility Stud. 2022 Sep 16;8:211. doi: 10.1186/s40814-022-01165-2 (PMC9479401; doi:10.1186/s40814-022-01165-2)
Supplement: Supplementary file 3 — Additional file 3: Appendix B. Baseline Questionnaire. [file 40814_2022_1165_MOESM3_ESM.pdf]

Default Question Block

Participant ID

[ ]

What year were you born in?

[ ]

What gender do you identify with? (Please select one)

- ○ Man
- ○ Woman
- ○ Two-spirit
- ○ Trans woman
- ○ Trans man
- ○ Non-binary (genderqueer)
- ○ [ ] Not listed
- ○ Don't know
- ○ Decline to answer

Do you identify with any of the racial identities listed below? (Please select all that apply)

- ☐ Arab (e.g., Syrian, Egyptian, Yemeni)
- ☐ Asian-East (e.g., Chinese, Korean, Japanese)

- ☐ Asian-South-East (e.g., Filipino, Vietnamese, Cambodian, Malaysian, Laotian)
- ☐ Asian-South or Indo-Caribbean (e.g., Indian, Pakistani, Sri Lankan, Indo-Guyanese, Indo-Trinidadian)
- ☐ Asian-West (e.g., Iranian, Afghan)
- ☐ Black-Canadian/American
- ☐ Black-African (e.g., Ghanaian, Ethiopian, Nigerian)
- ☐ Black-Afro-Caribbean or Afro-Latinx (e.g., Jamaican, Haitian, Afro-Brazilian)
- ☐ Indigenous (e.g., First Nations, Metis, Inuit)
- ☐ Latin American (e.g., Brazilian, Mexican, Chilean, Cuban)
- ☐ White (e.g., European, French, Ukrainian, Euro-Latinx)
- ☐ ________ Not listed (please specify)
- ☐ Don't know
- ☐ Decline to answer

Block 1

What kind of diabetes do you have? (Please select one)

- ○ Type 1: Often diagnosed in childhood/teenage years. Treated with insulin from the beginning.
- ○ Type 2: Often diagnosed later in adulthood. Treated with diet, exercise, medications and insulin (but not usually from the beginning).
- ○ Other: Includes but is not limited to diabetes related to pancreas damage (Type 3c such as from chronic pancreatitis, cystic fibrosis, hemochromatosis, pancreatic cancer, pancreas resection, etc.), MODY (maturity-onset diabetes of the young) and LADA (latent autoimmune diabetes in adults), and post-transplant diabetes mellitus. (Please specify) ________
- ○ Don't know

What age were you when you were diagnosed with diabetes?

When initially diagnosed, how was your diabetes managed? (Select all that apply)

- ☐ Diet
- ☐ Exercise
- ☐ Oral medications
- ☐ Insulin

How long was it from the time you were diagnosed until you started on insulin?

- ○ ____ Days
- ○ ____ Weeks
- ○ ____ Months
- ○ ____ Years

How many years have you had diabetes (from the time of your diagnosis until now)?

How do you currently manage your diabetes? (Please select all that apply)

- ☐ Diet
- ☐ Physical activity
- ☐ Medications (not insulin)
- ☐ Insulin (injections or pump)

Who do you currently see for management of diabetes and/or complications from your diabetes? (Please select all that apply)

- ☐ Family doctor/walk-in doctor
- ☐ Endocrinologist (doctor who specializes in diabetes)
- ☐ Internal medicine doctor
- ☐ Nephrologist (kidney doctor)
- ☐ Ophthalmologist (eye doctor; this is not the same as an optometrist)
- ☐ Diabetes nurse
- ☐ Registered dietitian
- ☐ Pharmacist
- ☐ Podiatrist (foot specialist)
- ☐ Foot care nurse

Block 2

Do you identify as having any substance use issues or addictions? "Past use" indicates that you have not used this substance in 6 months or longer.

	Current use	Past use (quit completely)	Never
a. Alcohol use disorder	○	○	○
b. Cannabis (marijuana) use disorder	○	○	○
c. Recreational or street drugs (including IV drugs)	○	○	○
d. Smoking (cigarettes)	○	○	○

Do you have any of the following conditions? You may have been told this by your doctor, have been started on treatment for this or referred to a specialized doctor for further management of this. (Please select all that apply)

- [ ] Depression
- [ ] Anxiety
- [ ] Bipolar disorder
- [ ] Psychosis (schizophrenia, schizoaffective disorder, delusional disorder, etc.)
- [ ] Personality disorder (borderline, histrionic, avoidant, schizoid, etc.)

Block 3

What is your current housing situation? (Please select all that apply)

- [ ] Own apartment/house
- [ ] Someone else's place
- [ ] Motel/hotel (self-funded)
- [ ] Hospital
- [ ] Treatment centre
- [ ] Jail, prison, remand centre
- [ ] Homeless shelter (emergency, family or domestic violence shelter)
- [ ] Motel/hotel (funded by city or homeless program)
- [ ] Transitional shelter/housing
- [ ] Unsheltered in a public place (e.g., street, park, bus shelter, forest or abandoned building)
- [ ] Encampment (e.g., group of tents, makeshift shelters or other long-term outdoor settlement)
- [ ] Vehicle (e.g., car, van, RV, truck, boat)

In the past 12 months, what was your housing situation? (Please select all that apply)

- [ ] Own apartment/house
- [ ] Someone else's place

- ☐ Motel/hotel (self-funded)
- ☐ Hospital
- ☐ Treatment centre
- ☐ Jail, prison, remand centre
- ☐ Homeless shelter (emergency, family or domestic violence shelter)
- ☐ Motel/hotel (funded by city or homeless program)
- ☐ Transitional shelter/housing
- ☐ Unsheltered in a public place (e.g., street, park, bus shelter, forest or abandoned building)
- ☐ Encampment (e.g., group of tents, makeshift shelters or other long-term outdoor settlement)
- ☐ Vehicle (e.g., car, van, RV, truck, boat)

In total, how long have you experienced homelessness? (Please select one)

- ○ ______ Length in days
- ○ ______ Length in weeks
- ○ ______ Length in months
- ○ ______ Length in years
- ○ Don't know
- ○ Decline to say

Default Question Block

Please read each statement and indicate to what degree you agree or disagree with each statement.

	Strongly Disagree	Disagree	A[illegible]
I have a good understanding of the health complications that can [illegible]	○	○	

result from diabetes.

	Strongly Disagree	Disagree	A
I have a good understanding of what screening for diabetes complications is recommended.	○	○	
I have a good understanding of why screening for diabetes complications is important.	○	○	
I see a diabetes doctor or nurse several time a year to review my diabetes management.	○	○	

Block 1

Please read each statement and indicate to what degree you agree or disagree with each statement.

	Strongly Disagree	Disagree
It is important to me to have my **blood work and urine tests** completed on a regular basis to see how well my diabetes is being managed.	○	○
It is easy for me to have my **blood work and urine tests** completed.	○	○
I have my **blood work and urine tests** completed every time I am given a requisition.	○	○

What are the challenges you face in getting your blood work and urine tests completed?

Block 2

Please read each statement and indicate to what degree you agree or disagree with each statement.

	Strongly Disagree	Disagr
It is important to me to have my annual screening **eye exam.**	○	○
It is easy for me to have my annual screening **eye exam** completed (dilated eye exam with an eye doctor).	○	○
I have my annual screening **eye exam** completed every year.	○	○

What are the challenges you face in getting your eye screening completed?

Block 4

Please read each statement and indicate to what degree you agree or disagree with each statement.

	Strongly Disagree	Disa
It is important to me to have my annual screening **foot exam** completed by a diabetes doctor or nurse.	○	○
It is easy for me to have my annual screening **foot exam.**	○	○
I have my screening **foot exam** completed every year.	○	○

What are the challenges you face in getting your foot screening completed?

Default Question Block

The following statements describe self-care activities related to your diabetes. Thinking about your self-care over the last 8 weeks, please specify the extent to which each statement applies to you.

	Applies very much	Applies t considera degree
I check my blood sugar levels with care and attention.	○	○
The food I choose to eat makes it easy to achieve optimal blood sugar levels.	○	○
I keep all doctors' appointments recommended for my diabetes treatment.	○	○
I take my diabetes medication (e.g., insulin, tablets) as prescribed.	○	○
Occasionally I eat lots of sweets or other foods rich in carbohydrates.	○	○
I record my blood sugar levels regularly (or analyze the value chart with my blood glucose meter).	○	○

	Applies very much	Applies t considera degree
I tend to avoid diabetes-related doctors' appointments.	○	○

	Applies very much	Applies t considera degree
[illegible]		
I do regular physical activity to achieve optimal blood sugar levels.		
I strictly follow the dietary recommendations given by my doctor or diabetes specialist.		
I do not check my blood sugar levels frequently enough as would be required for achieving good blood glucose control.		
I avoid physical activity, although it would improve my diabetes.		
I tend to forget to take or skip my diabetes medication (e.g., insulin, tablets).		

	Applies very much	Applies t considera degree
Sometimes I have real 'food binges' (not triggered by hypoglycaemia).		
Regarding my diabetes care, I should see my medical practitioner(s) more often.		
I tend to skip planned physical activity.		
My diabetes self-care is poor.		

Default Question Block

Which of the following diabetes issues are currently a problem for you? Select the option that gives the best answer for you. Please provide an answer for each question.

	Serious problem	Somewhat serious problem	Mo pro
Feeling scared when you think about living with diabetes.			
Feeling depressed when you think about living with diabetes.			
[illegible]			

	Serious problem	Somewhat serious problem	Mo[illegible] pro[illegible]
Worrying about the future and the possibility of serious complications.	○	○	
Feeling that diabetes is taking up too much of your mental and physical energy every day.	○	○	
Coping with complications of diabetes.	○	○	

Which medications (pills or injections that do not include insulin) do you take? (Please select all that apply)

- ☐ Metformin (Glucophage)
- ☐ Glipizide (Glucotrol)
- ☐ Gliclazide (Diamicron)
- ☐ Glyburide (Diabeta)
- ☐ Glibenclamide (Euglucon)
- ☐ Acarbose (Glucobay)
- ☐ Repaglinide (Gluconorm)
- ☐ Dapagliflozin (Forxiga)
- ☐ Empagliflozin (Jardiance)
- ☐ Canagliflozin (Invokana)
- ☐ Linagliptin (Trajenta/Jentadueto)
- ☐ Saxagliptin (Onglyza/Komboglyze)
- ☐ Sitagliptin (Januvia/Janumet)
- ☐ Pioglitazone (Actos)
- ☐ Rosiglitazone (Avandia/Avandamet)
- ☐ Liraglutide (Victoza)
- ☐ Semaglutide (Ozempic)
- ☐ Dulaglutide (Trulicity)
- ☐ Lixisenatide (Adlyxine)
- ☐ Unsure/don't know

Which of the following best describes your insulin pattern/regimen? (Please select one)

- Once daily long-acting insulin
- Twice daily long-acting insulin
- Twice daily pre-mixed insulin (long and short together)
- Fast-acting insulin with meals only
- Fast-acting insulin with meals and long-acting once daily
- Insulin pump therapy
- Other (please specify)
- Unsure/don't know

Please state if you have any of the following complications related to your diabetes. You may have been told this by your doctor, have been started on treatment for this or referred to a specialized doctor for further management of this.

	Yes	No
a. Cardiovascular disease (examples include coronary artery disease, myocardial infarctions or heart attacks)	◯	◯
b. Cerebrovascular disease (such as a stroke or mini stroke/TIA)	◯	◯
c. Peripheral vascular disease (this includes significant plaque build-up in the vessels of the arms and leg causing pain due to poor blood flow, or even requiring surgery to fix or bypass blockages in the vessels in the arms or legs, or even surgery to amputate a limb or portion of a limb)	◯	◯
d. Diabetic retinopathy (damage to the blood vessels in the eye, usually diagnosed by an eye doctor)	◯	◯
e. Diabetic nephropathy (damage to the kidneys related to your diabetes. This may result in poor kidney function or excessive protein being spilled into your urine due to the damage)	◯	◯
f. Diabetic neuropathy (damage to nerves related to diabetes. This may cause pain usually starting at the feet and moving up the legs and can cause loss of	◯	◯

[illegible] sensation) — Yes / No

g. Diabetic foot complications (infections, non-healing ulcers, deformity in the bones or joints) — ○ ○

Powered by Qualtrics
