# Supplementary material for: Developing and evaluating a SAFER model to screen for diabetes complications among people experiencing homelessness: a pilot study protocol
Source: Pilot Feasibility Stud. 2022 Sep 16;8:211. doi: 10.1186/s40814-022-01165-2 (PMC9479401; doi:10.1186/s40814-022-01165-2)
Supplement: Supplementary file 4 — Additional file 4: Appendix C. Follow-Up Questionnaire. [file 40814_2022_1165_MOESM4_ESM.pdf]

Default Question Block

Participant ID

Please read each statement and indicate to what degree you agree or disagree with each statement.

	Strongly Disagree	Disagree
I have a good understanding of the health complications that can result from diabetes.	○	○
I have a good understanding of what screening for diabetes complications is recommended.	○	○
I have a good understanding of why screening for diabetes complications is important.	○	○

Block 1

Please read each statement and indicate to what degree you agree or disagree with each statement.

	Strongly Disagree	Disagree
It is important to me to have my **blood work and urine tests** [illegible]	○	○

	Strongly Disagree	Disagree
completed on a regular basis to see how well my diabetes is being managed.	○	○
It was easy for me to have my **blood work and urine tests** completed at this visit.	○	○

Block 2

Please read each statement and indicate to what degree you agree or disagree with each statement.

	Strongly Disagree	Disagree
It is important to me to have my annual screening **eye exam.**	○	○
It was easy for me to have my annual screening **eye exam** completed at this visit.	○	○

Block 4

Please read each statement and indicate to what degree you agree or disagree with each statement.

	Strongly Disagree	Disa
It is important to me to have my annual screening **foot exam** completed by a diabetes doctor or nurse.	○	○
It was easy for me to have my annual screening **foot exam** at this visit.	○	○

Powered by Qualtrics
