# Supplementary material for: Developing and evaluating a SAFER model to screen for diabetes complications among people experiencing homelessness: a pilot study protocol
Source: Pilot Feasibility Stud. 2022 Sep 16;8:211. doi: 10.1186/s40814-022-01165-2 (PMC9479401; doi:10.1186/s40814-022-01165-2)
Supplement: Supplementary file 5 — Additional file 5: Appendix D. Interview Guides. [file 40814_2022_1165_MOESM5_ESM.docx]

# Interview Guide – Participants

Thank you for agreeing to participate in this interview. This should take 20-minutes and is aimed at exploring your personal experiences and feelings regarding your diabetes care. This is a confidential session and there are no wrong answers. We encourage your honest thoughts and opinions so that we can learn from them.

Interview Questions

**General**

1. What do you know about complications that can happen related to diabetes? (i.e., diabetes affects different parts of your body, do you know which parts it affects?)

**Screening**

1. Have doctors ever checked you for any of these complications before?
   1. For example: special diabetes eye test (with dilating drops), careful foot examination, or blood and urine tests to check on kidneys
   2. Please tell me about that experience
2. How often do you have each of those screening tests performed (e.g., once, once every few years, once a year etc.)? Why?
3. Can you walk me through a day where you are trying to get your diabetes eye exam completed?
   1. Where did you go to get this done?
   2. How easy or difficult was that for you to do?
   3. What were some of the barriers or challenges you faced to getting your eye exam completed?
      1. Prompts: transportation, time, visiting an unfamiliar office, cost
      2. Have you ever had any concerns or hesitations with going to a new office to see an eye doctor?
   4. In order to get your eye exam completed, were any sacrifices of other things required of you (due to cost, time or energy)?
   5. What would make it easier for you to have your eye exam completed?
4. Can you walk me through a day where you are trying to get your diabetes lab work (blood and urine tests) completed?
   1. Where did you go to get this done?
   2. How easy or difficult was that for you to do?
   3. What were some of the barriers or challenges you have faced to getting your lab work completed?
      1. Prompts: transportation, time, visiting an unfamiliar office, cost
      2. Have you ever had any concerns or hesitations with going to a new location to have your lab work completed?
   4. In order to get your lab work completed, were any sacrifices of other things required of you (due to cost, time or energy)?
   5. Are you usually able to get your diabetes lab work completed BEFORE your follow-up appointments with your doctor or nurse?
   6. What would make it easier for you to have your lab work completed ahead of your appointments?
5. Can you walk me through a day where you got your diabetes foot exam completed?
   1. Where did you go to get this done?
   2. How easy or difficult was that for you to do?
   3. What were some of the barriers or challenges you have faced to getting your foot exam completed?
      1. Prompts: transportation, time, visiting an unfamiliar office, cost
      2. Have you ever had any concerns or hesitations with going to a new location to have your foot exam completed?
   4. In order to get your foot exam completed, were any sacrifices of other things required of you (due to cost, time or energy)?
   5. What would make it easier for you to have your foot exam completed?
6. How often is realistic for you to see your diabetes health care specialist?
7. How easy is it to see your diabetes health care specialist?
8. What are some challenges or difficulties you face in seeing your diabetes health care specialist?
9. Can you describe how you felt the last time you accessed health care (at and outside of the DI)?

**Closing thoughts**

*Script: We want you to know that you have access to free eye screening every year. All you have to do is see an optometrist and let the optometrist know that you have diabetes. In addition, you should remind your health care provider to test your A1c (or average blood glucose) every three months and look at your feet and your kidney function once a year.*

1. Do you have any ideas how screening for diabetes complications – through lab work, eye exams, and foot exams – could be done in a way that is easier for you to access and complete?
   1. Where would the ideal location be for this to be provided?
   2. Would you continue attending the screening intervention offered here on a yearly basis if it became a permanent program?
