# Supplementary material for: Developing and evaluating a SAFER model to screen for diabetes complications among people experiencing homelessness: a pilot study protocol
Source: Pilot Feasibility Stud. 2022 Sep 16;8:211. doi: 10.1186/s40814-022-01165-2 (PMC9479401; doi:10.1186/s40814-022-01165-2)
Supplement: Supplementary file 6 — Additional file 6: Appendix E. Interview Guide – Health Care Providers. [file 40814_2022_1165_MOESM6_ESM.docx]

**Appendix E - Interview Guide – Health Care Providers**

Thank you for agreeing to participate in this interview. We would like to discuss your experience as health care providers and clinic site managers in participating in our intervention to improve access to diabetes screening tests for patients with diabetes who are experiencing homelessness. This should take approximately 30 minutes. This is a confidential session, and we appreciate your honesty and insights.

Interview Questions

1. What is your role in delivering this intervention to patients with diabetes who are experiencing homelessness?
2. What barriers do patients typically face in completing screening tests?
   1. What barriers prevent patients from completing diabetes foot exams?
   2. What barriers prevent patients from completing diabetes lab work (blood and urine testing)?
   3. What barriers prevent patients from completing diabetes screening eye exams?
   4. What barriers prevent patients from access care from other health care providers to manage diabetic complications (podiatry, ophthalmology, nephrology)?
3. From your perspective, prior to this intervention what barriers prevented you from fully assisting the target population in accessing or completing diabetes screening tests for patients experiencing homelessness and housing instability?
   1. What barriers prevented you from completing diabetes foot exams and referrals for foot care?
   2. What barriers prevented you from completing diabetes lab work (blood and urine testing) in patients?
   3. What barriers prevented you from assisting patients in completing diabetes screening eye exams and referrals for eye care?
4. If you are responsible for providing clinical care, prior to the intervention, how often did you feel you lacked enough information to make an optimal clinical decision for patients or to engage them in behavior change discussions during appointments?
5. From your perspective, did this intervention address the barriers that existed in accessing and completing diabetes screening tests?
   1. How did it improve your ability to assist in completing diabetes foot exams in patients?
   2. How did it improve your ability to assist in completing diabetes lab work (blood and urine testing) in patients?
   3. How did it improve your ability to assist in completing diabetes screening eye exams?
   4. How did it improve your ability to refer to other health care specialists upon detection of a diabetic complication?
6. What was your experience with implementing the intervention?
   1. Please explain whether the training or education you received was adequate in order to carry out the intervention?
   2. What impact did the intervention have on your ability to provide comprehensive care?
   3. Please comment on the ease of implementing the intervention into your clinical practice.
   4. What impact did the intervention have on your time or flow in clinic?
   5. How did the intervention influence your clinical decision making? (For example, did it lead to different decisions, hasten the clinical decision-making process, allow more time for discussion with patients to participate in shared decision making)
   6. Is there anything that could be added to this intervention to improve ease of screening for patients with diabetes who are experiencing homelessness
      1. Prompts: location of intervention, number of testing machines, number of individuals trained to use the testing machines
7. If you participated in utilizing the testing machines, please describe the experience?
   1. Describe your experience using the point-of-care A1C instrument
      1. Were there any unanticipated barriers to utilizing this instrument?
   2. Describe your experience using the point-of-care ACR instrument,
      1. Were there any unanticipated barriers to utilizing this instrument?
   3. Describe your experience using the handheld PanOptic ophthalmoscope and teleretinopathy program
      1. Were there any unanticipated barriers to utilizing this instrument?
   4. Describe your experience using the foot screening tool and referral pathway.
      1. Were there any unanticipated barriers to utilizing this instrument?
   5. Describe if there were ever any times where you were unable to perform the screening tests as described in the standardized operating procedure for the study.
      1. Prompts: equipment malfunction, lack of time, lack of space, etc.
8. Has this intervention impacted your confidence in being able to provide comprehensive care to patients with diabetes who are experiencing homelessness or your ability to assist these patients in accessing this care?
9. How would you feel if this intervention became usual practice?
   1. Is this intervention sustainable?
      1. What are some challenges to the sustainability of this intervention?
      2. Do you have any suggestions to make the intervention more likely to be maintained in practice?
   2. Does this intervention add value to the diabetes care in this population?

*Here a pilot trial means any randomised study conducted in preparation for a future definitive RCT, where the main objective of the pilot trial is to assess feasibility.
